# Vernalization, Photoperiod, and Gibberellin Coordinately Regulate Flower Bud Differentiation in *Oenothera biennis* L.

**DOI:** 10.3390/plants15142147

**Published:** 2026-07-12

**Authors:** Xiaoying Zhang, Zhiqin Guo, Yunting Chen, Pi Yin, Wuhua Zhang, Tao Yang, Jinzhu Zhang, Jie Dong

**Affiliations:** 1 College of Horticulture, Northeast Agricultural University, Harbin 150030, China; zxiaoying2013@163.com (X.Z.); 18045361673@163.com (Z.G.);; 2 Key Laboratory of Cold Region Landscape Plants and Applications, Harbin 150030, China; 3 Heilongjiang Yabuli Forestry Bureau Co., Ltd., Yabuli 150636, China

**Keywords:** facultative selfer, flowering regulation, flower bud differentiation, gibberellic acid, FT/FLC, *Oenothera biennis*, vernalization

## Abstract

*Oenothera biennis* L. is an economically important oil and ornamental crop. However, the regulatory mechanisms underlying its flower bud differentiation remain poorly understood. To elucidate the interactions between the environmental cues and molecular networks governing this process, this study investigated natural populations from the Lesser Khingan Mountains. The results demonstrate that exposure to 0–10 °C for at least 15 d and long-day (LD) conditions correlated strongly with floral induction. Notably, 200 mg·L^−1^ of exogenous GA_3_ effectively substituted for vernalization, significantly promoting bolting and advancing flower development. Expression profiling revealed an antagonistic expression pattern between floral activators such as *ObFT* and *ObAP2* and repressors such as *ObFLC*. Both vernalization and GA_3_ upregulated floral activators while suppressing the repressors, and these processes were coincident with the transition to flowering. Flower bud differentiation goes through six continuous stages, spanning from inflorescence primordia to gynoecium primordia formation. Hybrid fruit setting, the pollen–ovule ratio (98) and the outcrossing index (OCI = 3) confirmed that *O. biennisis* is a facultative selfer, with reproduction predominantly relying on self-pollination. This study provides a systematic characterization of the integrated regulatory network coordinating vernalization, photoperiod, and gibberellin in *Oenothera* flowering, proposing a framework for the putative FT-FLC antagonism. These findings establish a molecular foundation for the precision management of flowering time and informed breeding strategies.

## 1. Introduction

Evening primrose (*Oenothera biennis* L.) is a wild species native to eastern North America that has been cultivated as an oilseed crop in recent decades [1]. Widely introduced in China, it has since become naturalized across Northeast and North China, regions that now serve as the primary production areas for evening primrose seeds. The seeds contain 25.1% oil, of which γ-linolenic acid (an unsaturated fatty acid) accounts for 8.1% [2]. As biosynthetic precursors of anti-inflammatory mediators, such unsaturated fatty acids play a key role in maintaining the homeostasis and physiological functions of human tissues [3]. Furthermore, evening primrose exhibits remarkable drought tolerance and resistance to pests and diseases [4,5], alongside a capacity to thrive in soils contaminated with heavy metals [6]. These traits make it a promising candidate for the phytoremediation of mining areas [7]. Given that seed yield is closely linked to flower bud differentiation and flowering phenology, understanding the regulatory mechanisms governing these processes is essential for developing precise cultivation strategies.

Flowering marks the transition to sexual reproduction; thus, deciphering the environmental and endogenous signals that initiate floral bud differentiation is of paramount importance. Plant phenology is jointly influenced by intrinsic factors, such as genetic makeup, and extrinsic environmental cues, including photoperiod, temperature, and biotic interactions [8]. Inappropriate temperature and day length often result in a high incidence of biennial behavior in *O. biennis* when it is sown in spring or summer [9]. Vernalization, a critical physiological mechanism, directly governs the completion of floral induction, subsequently regulating tillering, number of leaves, inflorescence development, and, ultimately, yield formation [10]. However, taxonomic classification of *Oenothera* regarding its vernalization requirements (winter plants, spring plants, and semi-winter plants) remains contentious. Moreover, the increasing frequency of early-spring frost driven by global warming profoundly affects the cultivation timing of species requiring prolonged cold exposure [11]. This situation is analogous to observations made regarding winter wheat, where global warming inhibits vernalization, triggers early flowering, reduces the number of grains per spike, and shortens the filling period, leading to yield penalties [12,13]. *O. biennis* exhibits significant yield instability in response to climatic fluctuations. Consequently, defining the precise thermal thresholds and duration required for vernalization is crucial for optimizing planting schedules and ensuring stable yields.

Inductive environmental stimuli (e.g., photoperiod and ambient temperature) are perceived and transduced by endogenous integrators, driving the shoot apical meristem (SAM) from vegetative to reproductive growth. The FLOWERING LOCUS T (FT) protein serves as a central integrator, converging signals from diverse pathways, including photoperiod, vernalization, autonomic activity, and gibberellin (GA) signaling [14]. Conversely, FLOWERING LOCUS C (FLC) acts as a potent repressor of this transition, inhibiting key activators such as FT, SUPPRESSOR OF OVEREXPRESSION OF CO 1 (SOC1), and TWIN SISTER OF FT (TSF) [15]. *APETALA1* (*AP1*) is a floral meristem identity gene, and functions first to repress vegetative identity, then to help establish floral primordia, and finally to shape the differentiation of floral parts [16]. VERNALIZATION INSENSITIVE 3 (VIN3) serves as a reliable molecular marker for vernalization perception [17]. MSI1 is required for the efficient activation of CONSTANS (CO), thereby facilitating photoperiodic induction [18]. Epigenetic regulation is also critical. For instance, the *atxr7* mutant of *Arabidopsis* exhibits rapid flowering accompanied by down-regulated *FLC*, reduced H3K4me3, and enhanced H3K27me3 [19]. Exogenous GA_3_ has been shown to promote scape elongation and stimulate early-flowering of *Agapanthus praecox* [20], suggesting its potential to bypass vernalization requirements.

Although the morphology of flower bud differentiation is conserved, the specific timing and environmental thresholds vary significantly across species. Climate instability, particularly unpredictable spring temperatures, poses a substantial risk to cultivation. Conventional planting schedules may expose *O. biennis* to abrupt early-spring cold snaps, or premature high temperatures, potentially preventing the completion of vernalization. Therefore, elucidating the effects of temperatures, photoperiod and GA on flower bud differentiation is an important prerequisite for establishing precise agronomic practices. Intriguingly, the evolutionary trajectory of *Oenothera* is heavily influenced by its permanent translocation heterozygosity, which couples facultative selfing with alternate segregation to preserve superior genotypes [21]. While this genomic stability ensures phenotypic uniformity, it simultaneously restricts genetic recombination and adaptability. In the context of rapid climate change, the capacity for occasional outcrossing may be the key determinant of a lineage’s long-term survival. Identifying residual outcrossing potential in *O. biennis* is thus of dual importance: it offers insights into the species’ evolutionary resilience and provides a practical route to broaden the genetic base for crop improvement.

Previous studies have primarily focused on the cultivation techniques and economic value of evening primrose, yet the specific environmental thresholds, internal physiological responses, and core molecular mechanisms underlying its flower bud differentiation remain poorly characterized. In particular, how low temperature, photoperiod, and GA synergistically affect the flowering transition, as well as the specific roles of genes representing core pathways of flowering time in this network, urgently require systematic investigation. Using wild evening primrose from the Lesser Khingan Mountains as the experimental material, this study aims to comprehensively analyze the regulatory network of evening primrose flower bud differentiation by simulating vernalization, extended photoperiod, and GA_3_ treatments, combined with morphological observation, biochemical determination, and gene expression analysis. This research is expected to provide a scientific basis for the artificial manipulation of flowering time and the development of climate-resilient breeding strategies.

## 2. Results

### 2.1. A 15 d Low Temperature (0–10 °C) Was Necessary to Induce the Flowering of O. biennis

**Figure 1:**
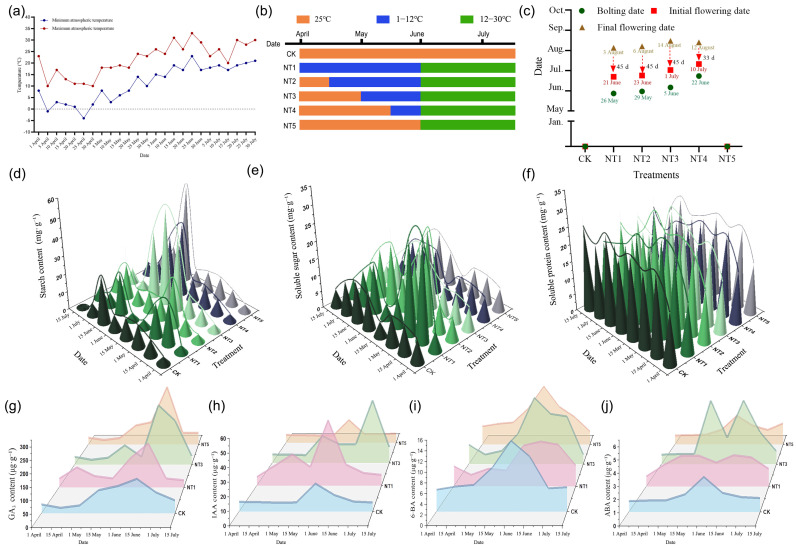
The effects of different temperature on the growth, development, carbon–nitrogen metabolism and endogenous hormone of *O. biennis*. (**a**) The daily maximum and minimum temperature during the experiment in Harbin. (**b**) Transplantation time in the open field. (**c**) The flowering period. (**d**) The starch content. (**e**) The soluble sugar content. (**f**) The soluble protein content. (**g**) The endogenous GA_3_ content. (**h**) The endogenous IAA content. (**i**) The endogenous 6-BA content. (**j**) The endogenous ABA content.

In Harbin, China, the maximum temperature (MaxT) during April was approximately 10 °C, while the minimum temperature (MinT) remained near 0 °C (Figure 1a). Evening primrose plants transplanted on 1 April (NT1) and 15 April (NT2) exhibited the earliest bolting and flowering times, along with the longest flowering duration (Figure 1b). Throughout May, the MaxT rose to approximately 15 °C, with the MinT ranging between 0 and 5 °C before 15 May. Plants transplanted on 1 May (NT3) initiated and terminated flowering later than NT1 and NT2, while their overall flowering duration remained consistent at approximately 45 d. For plants transplanted on 15 May (NT4), by which time the MinT had risen to approximately 10 °C, both the initial and final flowering times were further delayed compared to NT1 and NT2, and the flowering period was compressed to only 30 d. By June, when the MinT consistently exceeded 10 °C, plants transplanted at this time (NT5) failed to bolt or flower, displaying a vegetative phenotype similar to that of plants maintained in a heated greenhouse (CK) (Figure 1c). Furthermore, although a reduction in the duration of low-temperature treatment from NT1 to NT4 did not prevent the completion of the floral transition, it significantly shortened the period from bolting to flowering. Plants under the NT4 regime required only 18 d to flower post-bolting, substantially shorter than the 30 d observed for NT1 (Figure 1c). These findings indicate that MinT constrains the vernalization requirement of *O. biennis*, and that field-associated low-temperature exposure is a major determinant of its annual versus biennial habit.

### 2.2. Vernalization Determines Transition from Vegetative to Floral Buds of O. Biennis by Affecting Sugar and GA_3_ Levels

Owing to the influence of early spring low temperatures and flower bud formation, the starch and soluble sugar content in the NT1 and NT2 treatments exhibited bimodal patterns (Figure 1d,e, Tables S2 and S3). The peaks for NT1 occurred on 15 April and 1 June, whereas those for NT2 consistently lagged by 15 d relative to NT1. In contrast, the soluble sugar content in the other four treatments displayed only a single peak during the transition from vegetative to floral buds. Their starch content after 15 May was consistently higher than that in NT1 and NT2, whereas soluble sugar levels did not differ substantially from those in NT1 and NT2. The variation pattern of soluble protein content (Figure 1f, Table S4) was analogous to that of soluble sugars. These data indicate that earlier exposure to low temperature advanced the peak of energy metabolite accumulation in *O. biennis* shoot tips, and that starch hydrolysis to soluble sugars likely enhances cold resistance. Furthermore, sugars serve as signaling molecules involved in flowering-time regulation, and dynamic starch metabolism may contribute to both low-temperature adaptation and the indirect modulation of flowering.

Given the similarity in physiological indicator patterns between NT1 and NT2, as well as between NT3 and NT4, endogenous hormone levels were measured only for the CK, NT1, NT3, and NT5 treatments (Figure 1g–j, Tables S5–S8). In *O. biennis* plants treated with NT1 and NT3, the contents of IAA, GA_3_, and ABA in shoot apices exhibited two distinct peaks during floral bud differentiation and floral organ formation. Peaks in the NT3 treatment consistently appeared later than those in NT1. Conversely, in the non-flowering CK and NT5 treatments, hormone contents maintained relatively similar trends and levels, generally showing a single peak around 1 June. Notably, the GA_3_ content of all treatments exhibited a maximum on 15 June, with the value in the NT5 treatment even exceeding that of NT1. This likely reflects a plant response to environmental change (e.g., transplantation from a greenhouse to the open field) mediated through the adjustment in GA_3_ levels. Consequently, the elevated GA_3_ content induced by early low-temperature treatments in NT1 and NT3 appears to play a decisive role in floral bud differentiation of *O. biennis*.

### 2.3. Vernalization Can Be Substituted by Gibberellin to Promote the Flowering of O. biennis

**Figure 2:**
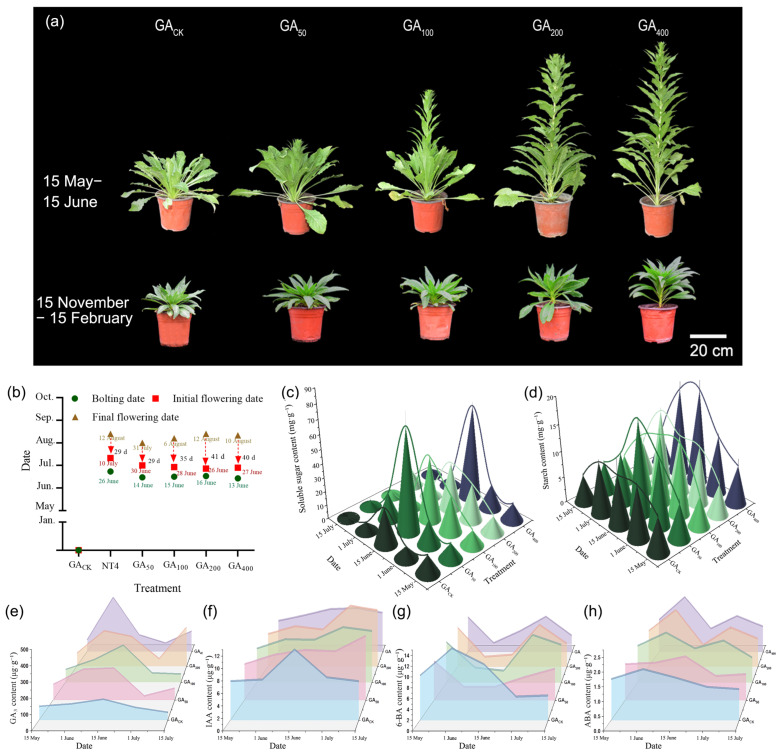
Effects of exogenous GA_3_ on growth, flowering time, carbohydrate metabolism, and endogenous hormone contents in *O. biennis*. (**a**) Effects of different concentrations of GA_3_ on *O. biennis*. (**b**) Flowering date. (**c**) The soluble sugar content. (**d**) The starch content. (**e**) The endogenous GA_3_ content. (**f**) The endogenous IAA content. (**g**) The endogenous 6–BA content. (**h**) The endogenous ABA content.

Evening primroses maintained in the greenhouse (CK) failed to bolt or flower. However, exogenous application of GA_3_ overcame this constraint. Applications initiated on 15 May significantly promoted vertical growth, with a concentration of 200 mg·L^−1^ of GA_3_ demonstrating the most pronounced effect (Figure 2a). Specifically, bolting height and floral development were markedly superior to those of the water-sprayed control (GA_CK_). Consequently, the initial flowering date was advanced by 14 d, and the flowering duration was extended from 29 d to 41 d (Figure 2b).

Following exogenous GA_3_ application, the starch and soluble sugar contents in all treatments initially increased before subsequently declining (Figure 2c,d, Tables S9 and S10), reaching a synchronous peak around 15 June. In stark contrast, the GA_ck_ group maintained persistently low levels of starch and soluble sugar, failing to reach the critical threshold required for floral bud differentiation and thereby preventing flower initiation. This indicates that exogenous GA_3_ bolstered starch accumulation and that elevated starch reserves are conducive to the transition from vegetative to reproductive growth. Subsequently, the gradual decline in soluble sugar suggests that GA_3_ enhanced carbohydrate transport and allocation, thereby supplying sufficient energy substrates for floral organ development in *O. biennis*.

Exogenous GA_3_ modulated endogenous GA_3_ level in the shoot apices of *O. biennis*. Endogenous GA_3_ exhibited a trimodal pattern, characterized by an initial increase, a subsequent decrease, and a later increase in the GA_50_, GA_200_, and GA_400_ groups, whereas it showed a single-peak in GA_100_ and GA_CK_ groups (Figure 2e, Table S11). In general, endogenous IAA levels across all exogenous GA_3_ treatments were higher than those in the GA_CK_ group (Figure 2f, Table S12). Moreover, exogenous GA_3_ caused a transient decrease in endogenous 6–BA content, followed by recovery and a subsequent decline (Figure 2g, Table S13), while the pattern of 6–BA content changes in the GA_CK_ group was the opposite. The endogenous ABA content exhibited two peaks, predominantly occurring on 1 June and 1 July in treatments in which the exogenous GA_3_ concentration reached or exceeded 100 mg·L^−1^ (Figure 2h, Table S14). The dynamics of these energy metabolites and hormones closely aligned with those observed under natural cultivation conditions, reinforcing the conclusion that exogenous GA_3_ mimics the effects of low-temperature vernalization.

### 2.4. Long-Day Photoperiodic Conditions Are Indispensable for the Growth of O. biennis

Spraying GA_3_ for 15 d starting from 15 May successfully induced bolting, flowering, and fruiting in greenhouse-cultivated plants. However, applying the same treatment on 15 November resulted in overall slow growth, significantly reduced plant height, and failure to flower (Figure 2a). Furthermore, seedlings pretreated at 4 °C or 10 °C with a 12 h photoperiod for one month, followed by field transplantation on 1 June, remained strictly vegetative and did not flower (Figure S1). These results demonstrate that floral bud differentiation in *O. biennis* requires not only vernalization but also LD photoperiodic conditions. Both factors are indispensable environmental cues for completing the life cycle and successfully transitioning to the reproductive phase.

### 2.5. O. biennis Completes Six Stages of Floral Bud Differentiation Within 24 d

**Figure 3:**
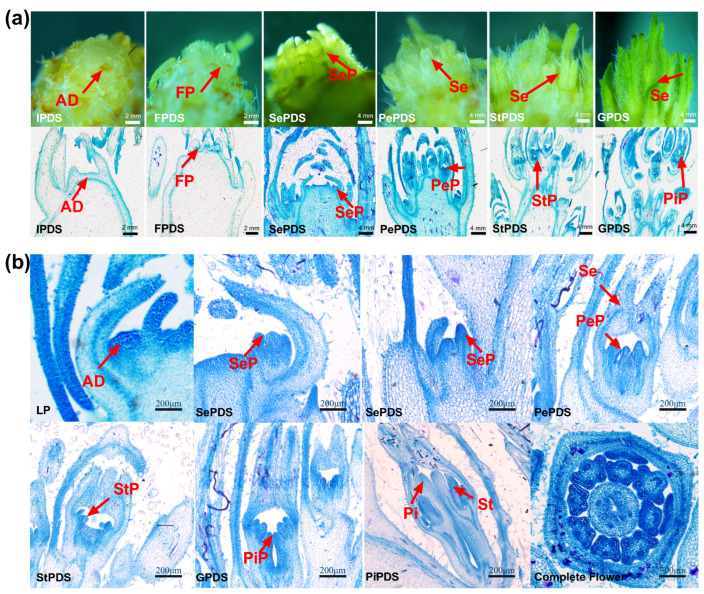
Morphological and histo-anatomical analysis of flower bud differentiation of *O. biennis*. (**a**) Flower bud differentiation morphology, (**b**) single flower differentiation anatomy. AD: Apical dome. DS: Differentiation stage. IP: Inflorescence primordia. FP: Floret primordia. Se: Sepal. PeP: Petal primordia. StP: Stamen primordia. GP: Gynoecium primordia. SeP: Sepal Primordia. PiP: Pistil Primordia. Pi: Pistil. LP: Leaf primordia.

The initiation of floral bud differentiation in *O. biennis* commenced in mid-May, marked by the formation of inflorescence primordia (IP). At this stage, the apical dome (AD) enlarged markedly, and assumed a hemispherical morphology. Epidermal cells were compact and small in volume and were enveloped by protective bracts (Figure 3a). Subsequently, the inflorescence axis elongated, generating multiple circular protrusions at the bract axils (Figure 3a,b), which corresponded to floret primordia (FP). The SAM elongated significantly, with concurrent increases in both height and width, signifying a definitive transition from vegetative to reproductive growth. Thereafter, the floret primordia invaginated to form arcuate structures, the apex of the AD transitioned from a hemispherical to a flattened morphology, and protrusions emerged at the periphery of the SAM, representing sepal primordia (SeP).

Petal primordia (PeP) emerged as protrusions at superior and medial positions relative to the sepals, and stamen primordia (StP) developed as two small bulges within the inner region of the petal primordia. Ultimately, the base of the flower expanded to form the gynoecium primordia (GP), whose distal extremity elongated into the style and stigma. At this juncture, the plant has bolted to approximately 10 cm in height, and the inflorescence structure was essentially established. The entire process, comprising six consecutive stages from IP initiation to GP formation, requires approximately 24 d. This morphogenetic progression exemplifies the precise temporal regulation underlying plant reproductive development.

### 2.6. Floral Bud Differentiation Involves Regulation by Multiple Pathway Genes in O. biennis

**Figure 4:**
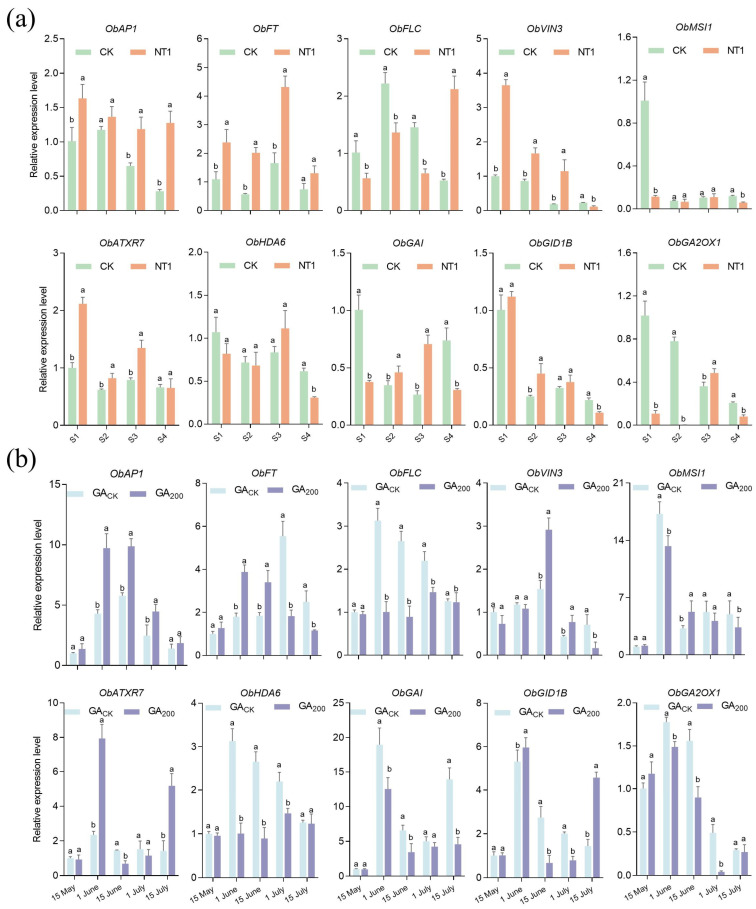
Effects of ambient temperature and exogenous GA_3_ application on the expression of flowering regulatory genes in evening primrose shoot tip. (**a**) Relative expression of ten genes of four stages under NT1 and CK treatment, respectively. S1: Flower bud differentiation, S2: floral development, S3: budding stage, S4: Initial flowering stage. (**b**) Relative expression of ten genes under 200 mg·L^−1^ of GA_3_ treatment and CK treatment, respectively. Different lowercase letters indicate significant differences among treatments (*p* < 0.05).

In the NT1 treatment, the expression levels of *ObAP1* and *ObFT* were significantly higher during the flower bud differentiation period (S1), floral organ development period (S2), budding stage (S3), and initial flowering stage (S4) than in the CK treatment, whereas the expression of *ObFLC* exhibited the opposite trend. From S1 to S2, plants that had undergone vernalization showed higher expression levels of *ObVIN3* and *ObATXR7* than greenhouse-grown plants. During the floral organ development period, the expression levels of *ObHDA6* and *ObGID1B* in the NT1 treatment were lower than those in CK. The relative expression levels of *ObGAI* and *ObGA2OX*, which negatively regulate gibberellin levels, were lower in the NT1-treated plants during the floral bud differentiation stage than in CK, which may partly explain the higher GA_3_ content observed in NT compared to CK on 15 June (Figure 4a). These results suggest that natural low temperatures in April and May promote flowering in *O*. *biennis* by up-regulating *ObAP1*, *ObFT*, *ObVIN3*, and *ObATXR7*, while suppressing the expression of *ObFLC*, *ObGAI*, and *ObGA2OX* during the floral bud differentiation period. Plants that had not undergone vernalization appeared to attempt to induce flowering via activation of LD pathway genes, but this effort was unsuccessful.

In the greenhouse, exogenous GA_3_, together with a LD photoperiod, increased the expression levels of *ObAP1*, *ObFT*, *ObVIN3*, and *ObATXR7* between 1 June and 15 June, while suppressing the levels of *ObFLC*, *ObGAI*, *ObGA2OX*, and *ObHDA6*, thereby enabling *O. biennis* to complete floral bud differentiation. During the blooming period, exogenous GA_3_ resulted in relative expression levels of *ObATXR7* and *ObGID1B* that were higher than those in CK, implying that these two genes also play a role during floral organ opening (Figure 4b).

### 2.7. Stigma Receptivity, Mating System, and Pollination Biology of Oenothera biennis: Insights into Facultative Autogamy

**Figure 5:**
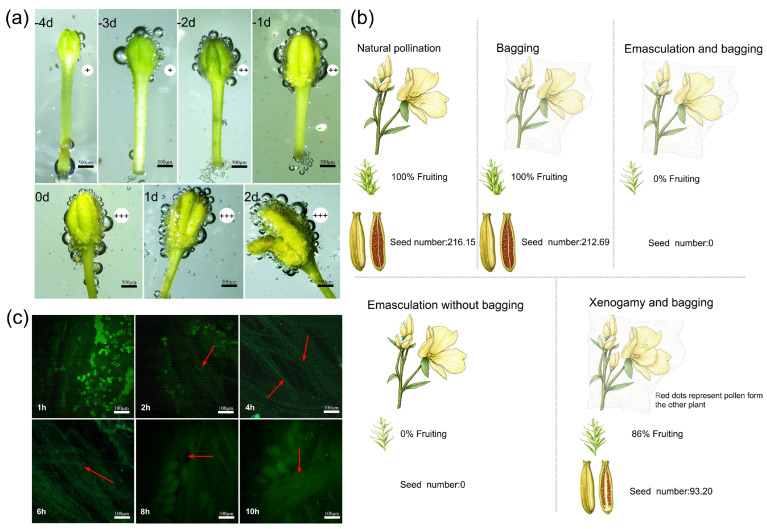
Stigma receptivity, mating system, and pollination biology of *O. biennis*. (**a**) Stigma receptivity of different flowering stages. (**b**) Fruit set and seed set of different pollination treatments. (**c**) Fluorescence microscopic observation of pollen tube growth at different pollination times. The red arrow represents the pollen tube.

In *O. biennis*, stigma receptivity began four days before petal opening. The intensity of stigma coloration, assessed by benzidine staining, progressively increased concomitantly with a rise in bubble formation around the stigma upon hydrogen peroxide infiltration. These observations indicate that stigma receptivity gradually intensified as flowering progressed (Figure 5a). Notably, even at the stage of petal wilting, i.e., two days after anthesis, the stigma retained substantial receptivity. *O. biennis* possesses eight yellow stamens. When the anther length reached 3~4 mm, the formation of male gametes via meiosis could be observed (Figure S2a). Stamen dehiscence occurred on the day preceding anthesis, clearly revealing the protogynous characteristic of this species, namely that pistils mature before stamens. During gynoecium development, the upper carpel fused to form the style and stigma, and the lower carpel enclosed the axial placenta and four locules, and the ovule primordium formed on the placenta (Figure S2b and Figure 3b).

In *O. biennis*, the outcrossing index (OCI) was 3, and each flower produced an average of 32,533 pollen grains and 332 ovules, resulting in a pollen–ovule ratio (P/O) of approximately 98, consistent with the characteristic range (31.9–396.0) for facultative selfing species. Seed setting following different pollination treatments revealed no significant difference between natural pollination (100% fruiting, and approximately 216.15 seeds) and bagged flowers (100% fruiting, and 212.69 seeds), both of which significantly exceeded the outcomes of xenogamy with bagging (86% fruiting, and 93.20 seeds) (Figure 5b). Moreover, emasculated flowers, whether bagged or unbagged, produced no fruit and seed.

Observation of pollen tube dynamics revealed that initial pollen tubes became visible on the stigma surface at 1 h post-pollination (Figure 5c). By 2 h, numerous pollen tubes had elongated rapidly along the style. They reached the middle region of the style at 4 h and extended to the style base at 6 h. By 8 h, pollen tubes had penetrated the ovary wall and approached the ovules, coinciding with the completion of fertilization. Active pollen tubes remained detectable at 10 h. These findings demonstrate that *O. biennis* exhibits typical characteristics of a facultative selfing plant, with no evidence of apomixis. Sexual reproduction primarily relies on self-pollination, although flowers retain the capacity to accept cross-pollen after anthesis. Cross-fertilization requires pollinators, but pollination efficiency remains low.

## 3. Discussion

### 3.1. Flower Bud Differentiation in Evening Primrose Is Jointly Regulated by Temperature, Long-Day Photoperiod, and Gibberellin

**Figure 6:**
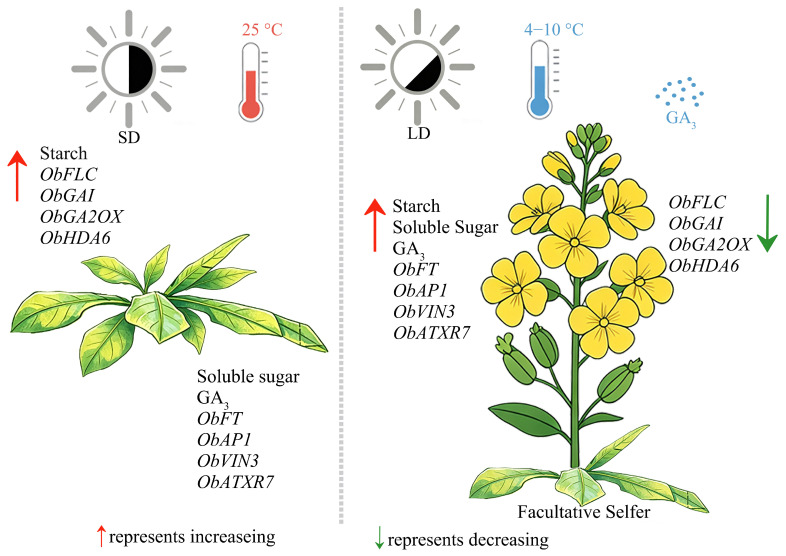
Photoperiod, vernalization and gibberellin affect floral transition of *O. biennis*.

*O. biennis* has an obligate LD photoperiod requirement for flowering and exhibits a facultative vernalization response [9]. Consistent with this, plants maintained in a heated greenhouse during winter failed to bolt or flower, irrespective of temperature (Figure 1). *O. biennis* is best characterized as a spring/summer-flowering LD plant, requiring approximately 15 d of exposure to temperatures ranging from 0 to 10 °C to synchronize reproductive development with seasonal cues. The defects in reproductive growth caused by the absence vernalization could be compensated for by exogenous gibberellin. However, under short-day conditions, plants remained in a vegetative state even after exogenous GA_3_ application, indicating that light signals are a prerequisite for initiating reproductive development (Figure 2 and Figure 6). This phenomenon is consistent with the finding that *Camellia oleifera* exhibits a significantly advanced flowering period under 16 h light exposure [22], but differs from the characteristics of poinsettia, which requires short-day induction for differentiation [23], highlighting species-specific photoperiod response mechanisms. However, the different transplanting dates (NT1–NT5) resulted in variations not only in temperature exposure but also in plant age, photoperiod, and the transition from greenhouse to open-field conditions. Consequently, although we observed a strong correlation between early-season low-temperature exposure (0–10 °C) and flowering, we cannot definitively isolate temperature as the sole causative factor. Similarly, the role of LD conditions was inferred from seasonal coincidence. Future studies employing growth chambers to control temperature and photoperiod independently are necessary to disentangle these effects and rigorously define the vernalization and photoperiodic requirements of *O. biennis*.

Some biennial plants such as oilseed rape and radish usually bloom in spring, while evening primrose blooms in summer. In addition to the LD for flower bud differentiation, rosette plants such as evening primrose grow until they reach a critical size for reproduction. This mechanism, which is associated with carbohydrate accumulation, ensures that individuals accumulate sufficient resources for successful reproduction [24]. Reducing the duration of light exposure causes the annual wild evening primrose (*O. mendoncinensis*) to bolt prematurely to ensure early seed production, but it also leads to a decrease in yield. The NT3 and NT4 treatments shortened vegetative period, thereby limiting carbohydrate stores available for reproductive growth, and yield. Although planting evening primrose on 15 April had little impact on seed yield, sowing or transplanting on 1 April is still recommended, as a longer growth period allows more nutrients to accumulate in rosette leaves and be transported to seeds, thereby protecting seed quality. Low-temperature treatment in wheat under low-light conditions is much less effective than under normal-light conditions. Similarly, evening primrose does not bloom under short-day conditions or when light exposure is inadequate [25].

Exogenous application of GA_3_ can replace vernalization to promote flowering in evening primrose, and this approach is commonly used in annual and biennial species, including biennial flowers. However, exogenous GA_3_ inhibited the expression of *EjFT2*, resulting in the failure of loquat tree to produce floral buds [26]. Gibberellin, a key plant hormone, can substitute for external environmental signals to induce flowering in annual and biennis species, with its regulatory mechanism exhibiting stage-specific characteristics [27]. Previous studies have shown that gibberellin advances scape emergence in *Clivia miniata* by 30 to 40 d [28] and promotes flower bud differentiation in lotus [29]. In contrast, GA inhibits floral transition in perennial woody plants, such as apple [30]. Floral buds in juvenile yellow camellias were induced by paclobutrazol, an inhibitor of GA biosynthesis [31]. The endogenous GA_3_ content during evening primrose flower bud development displayed characteristic fluctuations, following an “increase–decrease–increase–decrease” pattern. At the early stage of flower bud differentiation, GA_3_ levels increased significantly, a phenomenon similar to findings in peony [32], indicating that a relatively high GA_3_ concentration promotes the initiation of flower bud differentiation. As differentiation progressed, GA_3_ content gradually declined, suggesting that a low-concentration GA_3_ environment is more conducive to floral organ morphogenesis. This observation is consistent with conclusions from research on petunia [33].

This study confirms that gibberellin plays a definitive regulatory role in the flowering of evening primrose, and that the specific timing and amplitude of hormonal surges, rather than their mere presence, are critical for successful reproductive initiation. Moreover, the consistent bimodal peaks observed in the flowering treatments (NT1, NT3), in contrast to the unimodal patterns in the non-flowering controls (CK, NT5), provide robust biological evidence for the role of hormonal surges in mediating floral transition. Future studies quantifying the levels of these hormones at the cellular level within the SAM via HPLC-based metabolomics will further elucidate how these signals integrate with the epigenetic regulation of FT/FLC to determine the biennial versus annual habit.

### 3.2. Regulation of the Evening Primrose Flowering Network by the ObFT and ObFLC Genes

The flowering transition is regulated by an interconnected gene regulatory network, comprising both activators and inhibitors, that respond to internal and external stimuli during the flowering induction phase [34]. The floral transition in plants relies on the precise integration of multiple signaling pathways. *FT*, as a core floral integrator [35], receives inputs from the photoperiod, vernalization, gibberellin, and age pathways, and cooperates with inflorescence meristem-specific genes such as *AP1* to regulate the reproductive switch. In the absence of vernalization, FLC binds directly to the upstream of genes encoding flowering activators such as *FT* and *SOC1* and represses their transcription [36]. This study reveals for the first time that the central regulatory hub for flowering in evening primrose is the antagonistic ObFLC-ObFT module. Both natural temperature variation and exogenous gibberellin treatment significantly up-regulated the expression of *ObFT* and *ObAP1* while suppressing the flowering repressor *ObFLC*, indicating that this molecular switch is a key node for environmental signal transduction. This finding aligns with the classic model in *Arabidopsis thaliana*, in which *AtFLC* delays flowering by repressing *AtFT* expression. However, evening primrose exhibits unique regulatory features: vernalization suppresses *ObFLC* by inducing *ObVIN3* expression, whereas gibberellin directly inhibits *ObFLC* independent of low temperature to induce flowering (Figure 6).

Gibberellin signaling precisely regulated the flowering process in evening primrose through the *ObGID1b*-*ObGAI* module. Exogenous GA_3_ treatment significantly up-regulated the receptor gene *ObGID1b* while suppressing the negative regulator *ObGAI* and the metabolic gene *ObGA2OX1*, thereby creating a microenvironment with enhanced gibberellin signaling. This promoted flower bud differentiation, concurrently enhances *ObFT* expression, and inhibited the floral repressor *ObFLC*. In *Arabidopsis*, a key outcome of vernalization is the epigenetic silencing of the floral repressor *FLC* [37]. The expression of *ObMSI1* and *ObHDA6* remained stable under both treatments, indicating that the flowering regulation in evening primrose did not rely on the classical epigenetic silencing pathway. Although *ObATXR7*, a putative H3K4 methylation enzyme coding gene, was up-regulated under the treatment conditions, *ObFLC* was still effectively suppressed, suggesting the existence of an unknown epigenetic regulatory mechanism. The present study focused on a curated set of candidate genes based on conserved pathways in model species and identified strong correlative patterns between gene expression and flowering phenotypes. Nevertheless, the expression profiles and morphological staging presented here provide a robust foundation for selecting targets for future functional validation.

### 3.3. Flower Bud Differentiation, Pollination, and Compatibility in Spring-Blooming Plants

The breeding system of evening primrose exhibits characteristics of facultative selfing, as evidenced by its P/O ratio and OCI, which collectively indicate a selfing-dominated reproductive strategy. Pollen tube dynamics reveal that fertilization is completed within 8 h post-pollination. Combined with the onset of stigma receptivity three days before flowering and the phenomenon of precocious pollen maturity, this confirms the mechanism of self-pollination during the bud stage. This reproductive assurance strategy ensures stable seed setting in high-latitude habitats, contrasting with the variation in breeding systems observed in *Lycium ruthenicum* due to habitat differences [38]. Based on the bud development characteristics, the differentiation process of *P. pulcherrima* was divided into six stages as the initial differentiation stage, inflorescence primordium differentiation stage, flower primordium differentiation stage, sepal primordium differentiation stage, petal primordium differentiation stage, and column and pollen differentiation stage [39].

The developmental timeline of *O. biennis* is highly synchronized with both the protogyny and the self-pollination mechanism during the bud stage, reflecting an evolutionary adaptation for reproductive success. Self-pollination enables *Oenothera* to rapidly generate abundant, genetically uniform, and well-adapted progeny, thereby establishing dense pure-line populations. This reproductive mode effectively fixes and amplifies heterozygote advantage, accelerating the fixation and spatial expansion of superior genotypes. Although outcrossing yielded a relatively low fruit setting in this study (Figure 5), it permits gene flow within populations, introduces novel variation, and mitigates the risk of fitness decline associated with genetic uniformity. Collectively, these dual strategies underpin the spatiotemporal resilience of *Oenothera* populations (Figure 6). Nevertheless, permanent translocation heterozygosity imposes a rigid constraint on evolutionary potential: through the physical enforcement of alternate segregation and the genetic filter of balanced lethals, any creative potential afforded by outcrossing is strictly confined to the exchange of intact Renner complexes, rather than permitting widespread recombination or allelic reshuffling. This contrasts with the “best of both worlds” scenario observed in *Collinsia verna*, where both pollinator-delivered and autonomous self-pollen result in fruit production, suggesting that mixed mating is a likely outcome.

## 4. Materials and Methods

### 4.1. Plant Materials

The seeds of *O. biennis* were collected from natural populations in the Lesser Khingan Mountains, Heilongjiang Province, China, and were subsequently cultivated in Harbin. Air temperatures from early April to late July were obtained from The Weather Channel (https://weather.com/zh-CN/weather/today, accessed from 1 April 2022 to 30 July 2023) and are shown in Figure 1a.

### 4.2. Plant Treatment

Seeds were sown in the greenhouse on 1 March, and were transplanted to the fields on 1 April (NT1), 15 April (NT2), 1 May (NT3), 15 May (NT4), and 1 June (NT5), respectively. Plants cultivated in the greenhouse were used as the control group (CK) (Figure 1b). Shoot tips with young leaves were sampled every 15 d and stored at −80 °C, with the sampling period extending from 1 April to 1 August. For the constant low-temperature treatment: 60-day-old evening primrose seedlings were exposed on 15 May to 12 h light/12 h dark conditions at 4 °C (T4), 15 °C (T15), and 25 °C (T25) for 20 d, respectively. After a recovery period, the seedlings were transplanted outdoors, and sampling was conducted every 15 d until 1 August. Three replicates were performed for each treatment, with 30 plants in each replicate.

The control group was sprayed with distilled water, while the GA_3_ treatment included five concentration gradients: 50, 100, 150, 200, and 400 mg·L^−1^. GA_3_ was sprayed on the leaves of 30-day-old evening primrose seedlings, with 50 pots per treatment. Spraying was performed once every 3 d for a total of five applications. After treatment, sampling was carried out every 30 d by collecting the two innermost young leaves from the shoot tips together with the shoot tips themselves, which were then stored in liquid nitrogen for biochemical determination. Three replicates were performed for each treatment, with 30 plants in each replicate.

### 4.3. Determination of Macromolecular Nutrients and Endogenous Hormones

Soluble sugar and starch contents were determined using the anthrone colorimetric method [40] while soluble protein was quantitatively analyzed via Coomassie Brilliant Blue G-250 staining [41]. GA_3_, 6–BA, IAA, and ABA were measured using the enzyme-linked immunosorbent assay (ELISA) [42]. Three replicates were performed for each indictor, with 30 plants in each replicate.

### 4.4. Analysis of Gene Expression Levels

The two innermost young leaves adjacent to the shoot tip and the stem tip itself were collected as a mixed sample, wrapped in aluminum foil, and stored at −80 °C. RNA was extracted using the FastPure Plant Total RNA Isolation Kit (Vazyme, Nanjing, China) and subsequently stored at −80 °C. After RNA purity was assessed with a micro-volume UV spectrophotometer, RNA was reverse-transcribed into cDNA using the HiScript III 1st Strand cDNA Synthesis Kit (Vazyme, Nanjing, China). Based on previous transcriptomic data of evening primrose flower bud differentiation from our research group, relevant genes were selected and primers were designed (Table S1). These primers were synthesized by Sangon Biotech Co., Ltd. (Shanghai, China). *ObGAPDH* was chosen as the reference gene [7,43], and the expression levels of target genes in flower buds were quantified via real-time quantitative PCR. The qRT-PCR system and protocol were as described in the report by Zhang et al. [43]. Three biological replicates were included in the qRT-PCR analysis, each with three technical replicates. Gene expression levels were analyzed using the comparative Ct method [44]. Three replicates were performed for each indictor, with 30 plants in each replicate.

### 4.5. Observation of the Dynamic Process of Flower Bud Differentiation

From mid-May to the end of June, apical buds of *O. biennis* were collected from plants at 48 h intervals and immediately immersed in FAA fixative for storage at 4 °C. The samples underwent vacuum degassing treatment, and the fixative was replaced daily with fresh solution. After fixation, the samples were processed according to the standard paraffin sectioning procedure. Sections were cut at a thickness of 8 μm and stained with 0.5% toluidine blue for 10 min. The dynamic process of flower bud differentiation was systematically observed under an optical microscope (Eclipse E200, Nikon, Tokyo, Japan) to capture the typical morphological characteristics of each developmental stage. Ten flowers were examined at each stage.

### 4.6. Detection of Stigma Receptivity

Evening primrose flowers were collected at different flowering stages: 4 d before flowering, 3 d before flowering, 2 d before flowering, 1 d before flowering, on the day of flowering (0 d), and 1 d after flowering. Ten flowers were samples at each stage. Stigma receptivity was determined using the benzidine–hydrogen peroxide method.

### 4.7. Breeding System Inspection

The pollen–ovule ratio (P/O) of evening primrose was determined by calculating the ratio of total pollen grains per flower to the number of ovules per flower. This measurement was used to preliminarily assess the type of breeding system in evening primrose. The breeding system was further evaluated by measuring single-flower diameter, recording the temporal sequence of anther dehiscence and stigma receptivity, and measuring the relative spatial distance between the stamens (anthers) and pistil (stigma). The hybridization index was estimated by summing these three parameters. The assessment of the breeding system in evening primrose involved six pollination treatments: natural pollination (control), bagging without emasculation, artificial self-pollination within the same flower on the same plant, emasculation followed by bagging, emasculation with natural pollination, and emasculation with artificial cross-pollination using pollen from a different plant located 10 m away, respectively. Thirty plants were randomly selected as pollination samples in each treatment. The fruit setting rate and seed set rate were statistically analyzed.

### 4.8. Observation of the Meiosis Process in Microspores and Macrospores

Evening primrose anthers were collected during the bud stage and fixed in Carnoy’s fixative (anhydrous ethanol: glacial acetic acid = 3:1) at 4 °C for 24 h. the fixed specimens were rinsed three times with distilled water, and then hydrolyzed in 1 mol·L^−1^ HCl at 60 °C for 15 min. After another rinse with distilled water, surface moisture was blotted dry with filter paper, and the anthers were placed on microscope slides. A drop of Carbol Fuchsin stain was added to incubation for 15 min. A coverslip was gently pressed onto the slide to disperse the cells. The various stages of meiosis were systematically observed under an optical microscope (Eclipse E200, Nikon, Tokyo, Japan), and representative images were recorded.

### 4.9. Observation of Pollen Tube Growth Process

Following the method described by Soares [45], artificial pollination was performed on flower buds 2 d before flowering when anthers had not yet dehisced. Pistil samples were collected at 1, 2, 4, 6, 8, and 10 h post-pollination and fixed in FAA fixative for 24 h, respectively. At each time point, 10 pistils were randomly selected, rinsed three times with distilled water, softened in a 2 mol·L^−1^ NaOH solution at 60 °C for 25 min, and rinsed again with distilled water. Pistils were stained with water-soluble aniline blue solution (1 g·L^−1^ aniline blue + 1 g·L^−1^ dipotassium hydrogen phosphate) at 4 °C in the dark for 3 h. After staining, samples were placed on microscope slides, gently covered with coverslips to flatten the tissues, and observed under an upright fluorescence microscope (DS−RI2, Nikon, Tokyo, Japan) to track pollen tube growth dynamics. Pollen tube extension trajectories and distribution characteristics were recorded at each time point.

### 4.10. Statistical Analysis

Experimental data were organized and statistically analyzed using Excel. Analysis of variance was performed with SPSS 25.0, while data analysis and graphing were conducted using GraphPad Prism 8.02. All experiments included three independent biological replicates, and the results are expressed as the mean ± standard deviation. Statistical significance was assessed using ANOVA, with significance thresholds set at *p* < 0.05.

## 5. Conclusions

Floral initiation in *O. biennis* was strictly photoperiod-dependent; neither constant low temperatures (4 °C or 15 °C) for 15 d nor GA_3_ application under short-day conditions induced flowering. Conversely, LD conditions combined with exogenous GA_3_ effectively accelerated bolting, increased plant height, and advanced the flowering period by 14 d, with 200 mg·L^−1^ of GA_3_ identified as the optimal concentration. The floral bud differentiation in Heilongjiang occurs from mid-May, lasting 24 d and progressing through six distinct stages: inflorescence, floret, sepal, petal, stamen, and gynoecium primordia differentiation. *ObVIN3*, *ObAP1*, *ObFT*, *ObFLC*, *ObGA2OX1*, *ObGAI*, and *ObGID1b* constitute the core flowering pathway in *O. biennis*, while *ObMSI1*, *ObATXR7*, and *ObHDA6* likely play accessory roles. Furthermore, the species exhibits a mixed mating system, with stigma receptivity peaking three days pre-anthesis, pollen maturation four days pre-anthesis, and fertilization completing eight hours post-pollination. Reproduction occurs predominantly via selfing, while compatibility with outcrossing is retained. This comprehensive characterization provides a theoretical foundation for the genetic improvement and cultivation management of *O. biennis* in temperate regions.

## Data Availability

The raw data supporting the conclusions of this article will be made available by the authors on request.

## Supplementary Materials

The following supporting information can be downloaded at https://www.mdpi.com/article/10.3390/plants15142147/s1: Figure S1: Flowering of evening primrose under different temperature treatments; Figure S2: Meiosis process of pollen mother cells (a) and macrosporangia (b); Table S1: Primer list; Table S2: Changes of starch content of stem tips in *O. biennis* under different temperature treatments; Table S3: Soluble sugar content of stem tips in *O. biennis* under different temperature treatments; Table S4: Soluble protein content of stem tips in *O. biennis* under different temperature treatments; Table S5: Endogenous GA_3_ content of stem tips in *O. biennis* under different temperature treatments; Table S6: Endogenous 6-BA content of stem tips in *O. biennis* under different temperature treatments; Table S7: Endogenous IAA content of stem tips in *O. biennis* under different temperature treatments; Table S8: Endogenous ABA content of stem tips in *O. biennis* under different temperature treatments; Table S9: Starch content of stem tips in *O. biennis* under different GA_3_ concentration; Table S10: Soluble sugar content of stem tips in *O. biennis* under different GA_3_ concentration; Table S11: Endogenous GA_3_ content of stem tips in *O. biennis* under different GA_3_ concentration; Table S12: Endogenous IAA content of stem tips in *O. biennis* under different GA_3_ concentration; Table S13: Endogenous 6-BA content of stem tips in *O. biennis* under different GA_3_ concentration; Table S14: Endogenous ABA content of stem tips in *O. biennis* under different GA_3_ concentration.
