# Supplementary material for: Vernalization, Photoperiod, and Gibberellin Coordinately Regulate Flower Bud Differentiation in Oenothera biennis L
Source: Plants (Basel). 2026 Jul 12;15(14):2147. doi: 10.3390/plants15142147 (PMC13414904; doi:10.3390/plants15142147)
Supplement: Supplementary file 1 [file plants-15-02147-s001.zip › plants-4411869-supplementary.pdf]

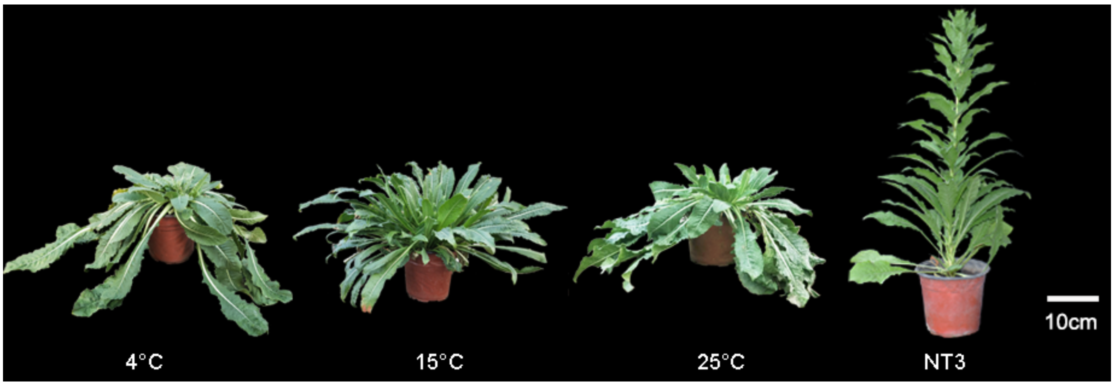

Figure S1 Flowering of evening primrose under different temperature treatments

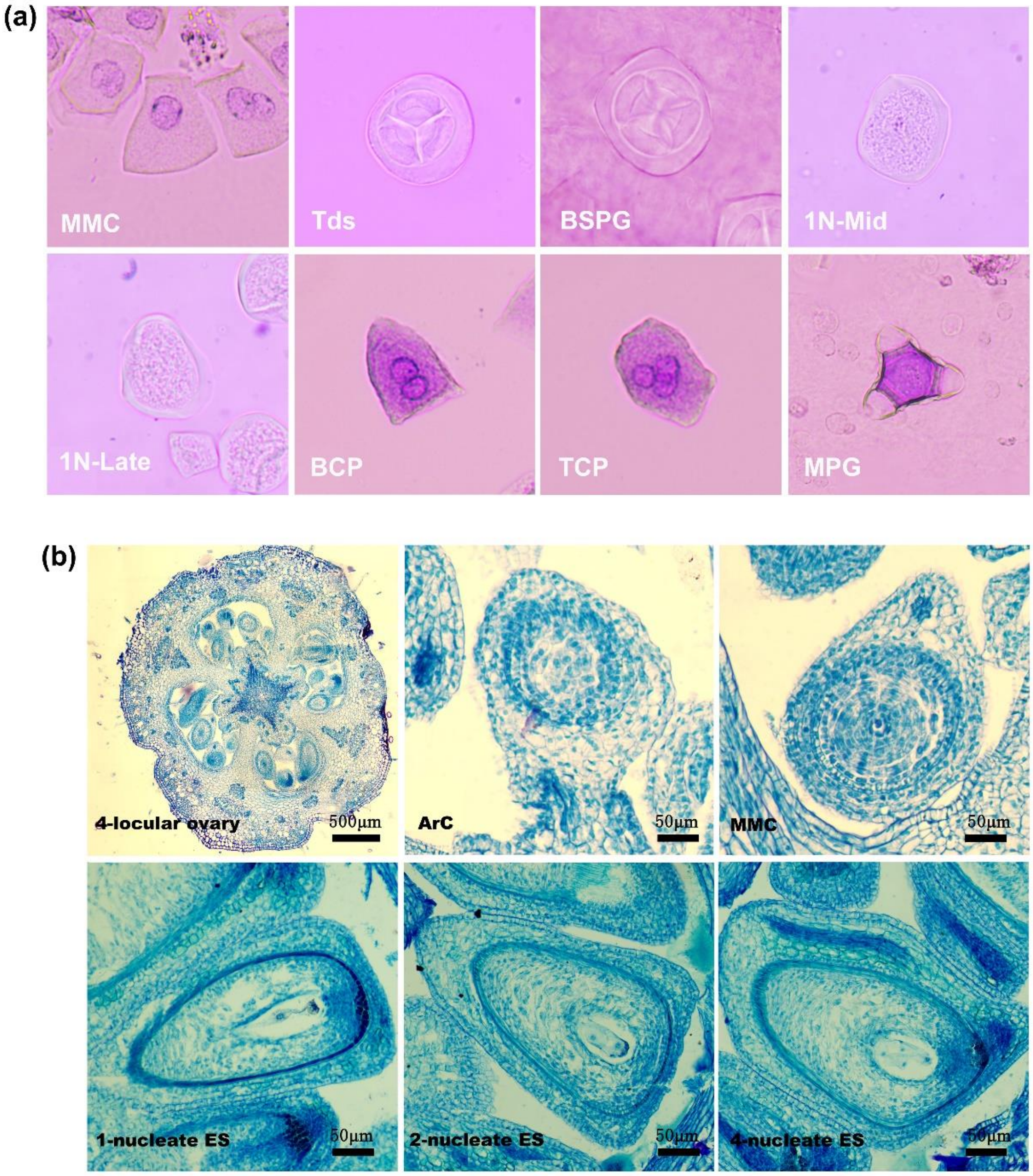

Figure S2 Meiosis process of pollen mother cells (a) and macrosporangia (b)

MMC: Megaspore mother cell (40×). Tds: Microspore tetrads (20×). BSPG: Bilaterally symmetrical pollen grain (20×). 1N-Mid: Mid-uninucleate stage (20×). 1N-Late: Late-uninucleate stage (20×). BCP: Bicellular pollen grain (20×). TCP: Tricellular pollen grain (20×). MPG: Mature pollen grain (20×). ArC: Archesporial Cell. MMC: Megasporocyte. 1-nucleateES: Uninucleate embryo sac. 2-nucleateES: Binucleate embryo sac. 4-nucleateES: Quadrinucleate embryo sac.

Table S1 Primer list

Primer name	Putative Ortholog (Arabidopsis)	Pathway Involved	Primer sequences (5'-3')
VIN3-F	Vernalization	Vernalization	TGGTACAGCAGAAGCCAAGA
VIN3-R	Insensitive 3		CTTCAGAGTGGCCCAAATCG
AP1-F	Apetala1	Floral meristem	TTCCCGGTGGGAAGGAATAC
AP1-R		marker gene	TGGAGCCTTCCTTTCAGGAG
MSI1-F	Multicopy	Photoperiod	ACGCCGTTTCTCTACGATCT
MSI1-R	Suppressor of Ira1		GCAAGCATCAGGTAGTTGGG
FLC-F	Flowering Locus	Floral Integrator	CGCAGACTGAAGAAAGCACA
FLC-R	C		CTGCTGGAGGAGACACAGAA
FT-F	Flowering Locus T	Floral Integrator	ACCCGTTGTGGAATGAGGAT
FT-R			GACGGGTTTATGGTCAAGCC
ATXR7-F	Histone-Lysine N-	Epigenetic modification	TGAGTCAGTTTCGGAGGCTT
ATXR7-R	Methyltransferase ATXR7		ATCTCACAAGCAGGCTCAGT
HDA6-F	Histone	Epigenetic modification	GACATTGGTGCAGGCTTAGG
HDA6-R	Deacetylase 6		ATCCGCTCCACACTGAAGAA
GID1b-F	Gibberellin	Gibberellin signaling	CAACACCTGGGTCCTCATCT
GID1b-R	receptor GID1B		CTGCTAGGTCGCGATTGAAG
GA2OX1-F	Gibberellin 2-beta-	Gibberellin biosynthesis	GCTTCCGAAGATCGAATCGG
GA2OX1-	dioxygenase 1		GGTACCGAAATCCAGTTGCC

R				
GAI-F	DELLA	protein	Gibberellin signaling	CAGTGAGGACCTGGTGATGT
GAI-R	GAI			AACGCATTGGAACCGAGATG
GAPDH-F				ACGAACCTCTTGTCTCGGTT
GAPDH-R				AAGTGTGCCAAATCCACCAC

Table S2 Changes of starch content of stem tips in *O. biennis* under different temperature treatments

	CK	NT1	NT2	NT3	NT4	NT5
Apr. 1st	4.36±0.29a	4.31±0.55a	4.36±0.29a	4.36±0.29a	4.36±0.29a	4.36±0.29a
Apr. 15th	8.86±2.42b	23.55±1.87a	8.86±2.42b	8.86±2.42b	8.86±2.42b	8.86±2.42b
May. 1st	12.29±0.62c	18.86±0.41b	26.9±0.45a	12.29±0.62c	12.29±0.62c	12.29±0.62c
May. 15th	11.90±1.46b	4.97±1.28c	11.87±0.67b	42.02±2.42a	11.9±1.46b	11.90±1.46b
Jun. 1st	9.14±2.03c	4.30±0.53d	11.3±0.71c	53.78±3.36a	40.26±0.93b	9.14±2.03c
Jun. 15th	26.69±1.62d	23.31±0.27e	8.57±2.02f	37.79±1.31b	29.62±0.83c	54.64±1.32a
Jul. 1st	6.88±1.47c	8.15±0.45c	19.8±9.12a	5.06±1.65c	11.11±1.81bc	16.03±1.25ab
Jul. 15th	0.65±0.41c	7.02±0.92b	7.93±0.6ab	8.40±1.52ab	7.17±1.58b	9.44±1.75a

Note: Statistical significance was assessed using ANOVA, with significance thresholds set at $p < 0.05$, N=90. Different lowercase letters indicate significant differences among treatments at the same time point.

Table S3 Soluble sugar content of stem tips in *O. biennis* under different temperature treatments

	CK	NT1	NT2	NT3	NT4	NT5
Apr. 1st	6.91±0.54a	6.00±0.47a	6.91±0.54a	6.91±0.54a	6.91±0.54a	6.91±0.54a
Apr. 15th	8.64±1.40b	29.37±2.26a	8.64±1.40b	8.64±1.40b	8.64±1.40b	8.64±1.40b
May. 1st	8.86±0.34b	27.68±1.21a	28.02±0.85a	8.86±0.34b	8.86±0.34b	8.86±0.34b
May. 15th	4.69±0.01d	8.81±0.50c	17.27±0.58a	12.94±3.41b	4.69±0.01d	4.69±0.01d
Jun. 1st	10.24±0.62c	4.69±0.59d	10.3±0.79c	17.68±1.29a	13.31±1.04b	10.24±0.62c
Jun. 15th	12.25±1.20b	15.07±0.99b	18.42±3.89a	14.65±0.52b	13.51±0.42b	14.32±0.35b
Jul. 1st	10.11±0.49c	10.84±0.23c	14.89±0.51a	10.23±0.34c	12.42±0.42b	12.50±0.33b
Jul. 15th	5.44±0.45b	5.86±0.28b	7.90±2.12a	5.14±0.68b	4.37±0.45b	4.07±1.04b

Note: Statistical significance was assessed using ANOVA, with significance thresholds set at $p < 0.05$, N=90. Different lowercase letters indicate significant differences among treatments at the same time.

Table S4 Soluble protein content of stem tips in *O. biennis* under different temperature treatments

	CK	NT1	NT2	NT3	NT4	NT5
Apr. 1st	15.36±1.14a	14.95±0.97a	15.36±1.14a	15.36±1.14a	15.36±1.14a	15.36±1.14a
Apr. 15th	23.94±1.52a	26.08±1.54a	23.94±1.52a	23.94±1.52a	23.94±1.52a	23.94±1.52a
May. 1st	22.36±1.43b	28.42±1.31a	26.52±0.55a	22.36±1.43b	22.36±1.43b	22.36±1.43b
May. 15th	24.54±0.42bc	23.89±0.22c	25.26±0.54b	25.54±1.01a	24.54±0.42bc	24.54±0.42bc
Jun. 1st	22.21±1.39c	25.90±0.39b	30.05±0.97a	27.58±2.57a	21.60±1.30c	22.21±1.39c
Jun. 15th	26.64±0.61a	21.35±3.26b	22.93±1.51b	17.23±1.77c	21.06±1.41b	20.66±1.08b
Jul. 1st	21.81±1.16b	19.67±0.8bc	19.52±1.35bc	18.46±1.23cd	16.78±2.80d	24.82±0.25a
Jul. 15th	25.09±2.50a	18.32±1.75b	21.71±3.20ab	21.07±1.21ab	21.99±2.91ab	18.36±0.82b

Note: Statistical significance was assessed using ANOVA, with significance thresholds set at $p < 0.05$, N=90. Different lowercase letters indicate significant differences among treatments at the same time.

Table S5 Endogenous GA_3 content of stem tips in *O. biennis* under different temperature treatments

Date	CK	NT1	NT3	NT5
Apr. 1st	34.38±3.22a	36.64±4.59a	34.48±4.24a	32.1±3.44a
Apr. 15th	20.75±1.97b	82.32±3.82a	20.75±1.97b	20.75±1.97b
May. 1st	30.05±0.47b	46.18±1.13a	30.05±0.47b	30.05±0.47b
May. 15th	88.91±7.11a	36.75±4.12b	84.44±1.86a	88.91±7.11a
Jun. 1st	104.76±4.26b	120.19±4.33a	34.4±1.63c	104.76±4.26b
Jun. 15th	132.42±3.70c	181.03±22.38b	261.61±18.98a	270.23±1.11a
Jul. 1st	80.39±5.64b	36.51±2.44d	193.18±2.35a	55.70±2.03c
Jul. 15th	49.51±6.42a	29.17±2.03b	37.65±5.32b	54.76±5.64a

Note: Statistical significance was assessed using ANOVA, with significance thresholds set at $p < 0.05$, N=90. Different lowercase letters indicate significant differences among treatments at the same time point.

Table S6 Endogenous 6-BA content of stem tips in *O. biennis* under different temperature treatments

Date	CK	NT1	NT3	NT5
Apr. 1st	4.26±0.04ab	4.21±0.02c	4.31±0.02a	4.26±0.04ab
Apr. 15th	4.86±0.06a	2.38±0.10b	2.09±0.06c	4.86±0.06a
May. 1st	5.18±0.03a	3.66±0.03b	2.92±0.03c	5.18±0.03a
May. 15th	8.56±0.46a	3.29±0.13c	6.68±0.08b	8.56±0.46a
Jun. 1st	13.76±0.11b	8.46±0.13c	14.72±0.09a	13.76±0.11b
Jun. 15th	10.69±0.48a	9.31±0.14b	11.11±0.22a	8.92±0.08b
Jul. 1st	4.51±0.04d	8.64±0.07b	10.54±0.10a	6.51±0.16c
Jul. 15th	4.73±0.03a	4.36±0.06b	2.08±0.04d	3.12±0.20c

Note: Statistical significance was assessed using ANOVA, with significance thresholds set at $p < 0.05$, N=90. Different lowercase letters indicate significant differences among treatments at the same time point.

Table S7 Endogenous IAA content of stem tips in *O. biennis* under different temperature treatments

Date	CK	NT1	NT3	NT5
Apr. 1st	6.87±0.03a	6.78±0.03b	6.87±0.03a	6.85±0.04a
Apr. 15th	6.67±0.06b	15.13±0.19a	6.67±0.06b	6.67±0.06b
May. 1st	6.28±0.01b	23.8±0.22a	6.28±0.01b	6.28±0.010b
May. 15th	6.43±0.04c	14.22±0.20b	21.81±0.19a	6.43±0.04c
Jun. 1st	19.85±0.81b	50.17±0.74a	15.2±0.59c	19.85±0.81b
Jun. 15th	11.69±0.26c	16.05±0.13a	15.15±0.20b	7.53±0.07d
Jul. 1st	7.06±0.10c	9.57±0.19b	51.09±2.13a	7.47±0.15c
Jul. 15th	6.42±0.04c	8.16±0.07b	12.73±0.37a	8.01±0.12b

Note: Statistical significance was assessed using ANOVA, with significance thresholds set at $p < 0.05$, N=90. Different lowercase letters indicate significant differences among treatments at the same time point.

Table S8 Endogenous ABA content of stem tips in *O. biennis* under different temperature treatments

Date	CK	NT1	NT3	NT5
Apr. 1st	0.85±0.01a	0.85±0a	0.86±0.01a	0.86±0.01a
Apr. 15th	0.89±0b	1.8±0.04a	0.89±0b	0.89±0b
May. 1st	0.91±0.01b	2.58±0.05a	0.91±0.01b	0.91±0.01b
May. 15th	1.38±0.04c	2.59±0.04b	5.72±0.28a	1.38±0.04c
Jun. 1st	2.74±0.08a	2.00±0.03c	2.34±0.13b	2.74±0.08a
Jun. 15th	1.49±0.02d	2.62±0.03b	5.73±0.12a	1.81±0.05c
Jul. 1st	1.16±0.02d	2.44±0.13b	2.86±0.08a	1.38±0.04c
Jul. 15th	1.09±0.03c	1.47±0.05b	1.14±0.03c	2.01±0.12a

Note: Statistical significance was assessed using ANOVA, with significance thresholds set at $p < 0.05$, N=90. Different lowercase letters indicate significant differences among treatments at the same time.

Table S9 Starch content of stem tips in *O. biennis* under different GA_3 concentration

	GA_{ck}	GA_{50}	GA_{100}	GA_{200}	GA_{400}
May 15th	13.71±1.58a	11.9±1.46a	11.43±1.81a	12.42±2.54a	12.91±1.04a
Jun. 1st	9.14±2.03c	18.17±1.16b	26.81±1.10a	26.79±2.23a	17.70±1.04b
Jun. 15th	26.69±1.62c	73.13±9.73a	45.91±1.36b	33.92±0.16c	66.55±2.74a
Jul. 1st	6.88±1.47b	19.38±1.23a	8.43±2.29b	8.35±2.31b	7.53±1.49b
Jul. 15th	1.04±0.18c	4.95±1.89b	4.23±0.34b	12.05±1.10a	5.10±0.62b

Note: Statistical significance was assessed using ANOVA, with significance thresholds set at $p < 0.05$, N=90. Different lowercase letters indicate significant differences among treatments at the same time.

Table S10 Soluble sugar content of stem tips in *O. biennis* under different GA_3 concentration

	GA_{ck}	GA_{50}	GA_{100}	GA_{200}	GA_{400}
May 15th	6.85±0.73a	6.91±0.88a	5.96±0.29a	6.05±1.22a	7.54±1.34a
Jun. 1st	10.24±0.62b	13.18±0.21a	13.64±0.64a	11.13±0.56b	11.22±0.59b
Jun. 15th	9.86±3.27c	17.95±0.28a	13.32±1.15b	11.35±1.24bc	18.16±1.67a
Jul. 1st	10.12±0.49c	9.79±0.29c	11.28±0.76c	14.25±0.55b	16.71±1.41a
Jul. 15th	5.43±0.45b	6.03±0.34ab	5.34±0.24b	6.08±1.08ab	6.71±0.26a

Note: Statistical significance was assessed using ANOVA, with significance thresholds set at $p < 0.05$, N=90. Different lowercase letters indicate significant differences among treatments at the same time.

Table S11 Endogenous GA_3 content of stem tips in *O. biennis* under different GA_3 concentration

	GA_{ck}	GA_{50}	GA_{100}	GA_{200}	GA_{400}
May 15th	88.37±7.61b	106.30±4.58a	91.86±8.82ab	99.23±5.87ab	93.55±9.14ab
Jun. 1st	102.89±3.82e	207.96±28.83c	160.85±1.46d	264.46±1.64b	427.87±1.62a
Jun. 15th	132.42±3.70c	215.49±0.54b	262.7±3.16a	221.50±2.36b	132.31±5.30c
Jul. 1st	80.39±5.64a	28.55±4.06d	66.97±0.34b	56.94±0.97c	69.69±0.68b
Jul. 15th	49.51±6.42d	80.31±0.91c	59.14±0.90d	293.95±7.27a	162.21±20.37b

Note: Statistical significance was assessed using ANOVA, with significance thresholds set at $p < 0.05$, N=90. Different lowercase letters indicate significant differences among treatments at the same time.

Table S12 Endogenous IAA content of stem tips in *O. biennis* under different GA_3 concentration

	GA_{ck}	GA_{50}	GA_{100}	GA_{200}	GA_{400}
May 15th	6.43±0.04a	6.35±0.03b	6.29±0.02b	6.34±0.05b	6.35±0.03b
Jun.1st	6.70±0.05d	7.97±0.07a	7.98±0.09a	7.81±0.09b	7.49±0.06c
Jun.15th	11.69±0.26a	8.81±0.08b	7.88±0.02c	7.17±0.03d	8.76±0.08b
Jul.1st	7.06±0.10e	8.57±0.22d	10.2±0.18b	11.83±0.08a	8.97±0.03c
Jul.15th	6.42±0.04e	11.27±0.14a	9.51±0.18c	10.95±0.18b	8.01±0.12d

Note: Statistical significance was assessed using ANOVA, with significance thresholds set at $p < 0.05$, N=90. Different lowercase letters indicate significant differences among treatments at the same time.

Table S13 Endogenous 6-BA content of stem tips in *O. biennis* under different GA_3 concentration

	GA_{ck}	GA_{50}	GA_{100}	GA_{200}	GA_{400}
May 15th	8.56±0.46a	8.01±0.12b	8.71±0.17a	8.88±0.22a	8.40±0.10ab
Jun. 1st	13.76±0.11a	2.75±0.08c	3.24±0.06b	2.26±0.01d	2.26±0d
Jun. 15th	10.69±0.48a	2.74±0.02c	2.56±0.04c	2.73±0.02c	5.02±0.02b
Jul. 1st	4.51±0.04d	4.73±0.24d	10.22±0.25a	9.43±0.03b	8.45±0.04c
Jul. 15th	4.73±0.03d	6.44±0.42b	8.09±0.12a	5.19±0.03c	3.97±0e

Note: Statistical significance was assessed using ANOVA, with significance thresholds set at $p < 0.05$, N=90. Different lowercase letters indicate significant differences among treatments at the same time.

Table S14 Endogenous ABA content of stem tips in *O. biennis* under different GA_3 concentration

	GA_{ck}	GA_{50}	GA_{100}	GA_{200}	GA_{400}
May 15th	1.44±0.02ab	1.35±0.04c	1.44±0.06ab	1.48±0.01a	1.40±0.02bc
Jun. 1st	1.79±0.04c	1.40±0.05d	1.96±0.05b	2.33±0.10a	2.40±0.03a
Jun. 15th	1.49±0.02b	1.63±0.01a	1.34±0.01c	0.90±0.01e	1.03±0.01d
Jul. 1st	1.16±0.02c	0.91±0.01d	1.65±0.04a	1.61±0.06b	1.57±0.01b
Jul. 15th	1.09±0.03ab	0.97±0.05b	0.98±0.02b	1.15±0.08a	1.12±0.10a

Note: Statistical significance was assessed using ANOVA, with significance thresholds set at $p < 0.05$, N=90. Different lowercase letters indicate significant differences among treatments at the same time.
